# Enhancer RNA-based modeling of adverse events and objective responses of cancer immunotherapy reveals associated key enhancers and target genes

**DOI:** 10.3389/fonc.2022.1048127

**Published:** 2023-01-19

**Authors:** Mengbiao Guo, Zhiya Lu, Yuanyan Xiong

**Affiliations:** ^1^ Key Laboratory of Gene Engineering of the Ministry of Education, Institute of Healthy Aging Research, School of Life Sciences, Sun Yat-sen University, Guangzhou, China; ^2^ Department of Medical Research Center, Sun Yat-sen Memorial Hospital, Guangzhou, China

**Keywords:** enhancer RNA (eRNA), immune checkpoint block therapy, adverse effect, drug responses, TCGA, pan-cancer analysis, TMEM43/LUMA

## Abstract

Immune checkpoint inhibitors (ICI) targeting PD-1/PD-L1 or CTLA-4 are emerging and effective immunotherapy strategies. However, ICI-treated patients present heterogeneous responses and adverse events, thus demanding effective ways to assess benefit over risk before treatment. Here, by integrating pan-cancer clinical and molecular data, we tried to predict immune-related adverse events (irAEs, risk) and objective response rates (ORRs, benefit) based on enhancer RNAs (eRNAs) expression among patients receiving anti-PD-1/PD-L1 therapies. We built two tri-variate (eRNAs) regression models, one (with ENSR00000326714, ENSR00000148786, and ENSR00000005553) explaining 71% variance (R=0.84) of irAEs and the other (with ENSR00000164478, ENSR00000035913, and ENSR00000167231) explaining 79% (R=0.89) of ORRs. Interestingly, target genes of irAE-related enhancers, including upstream regulators of MYC, were involved in metabolism, inflammation, and immune activation, while ORR-related enhancers target *PAK2* and *DLG1* which participate in T cell activation. More importantly, we found that ENSR00000148786 probably enhanced TMEM43/LUMA expression mainly in B cells to induce irAEs in ICI-treated patients. Our study provides references for the identification of immunotherapy-related biomarkers and potential therapeutic targets during immunotherapy.

## Introduction

Immune checkpoints (ICs) generally refer to key inhibitory factors of the immune system, including programmed cell death 1 (PD-1 or CD279) and its ligand programmed cell death 1 ligand 1 (PD-L1 or CD274) that control the T cell response and fate during tumor immunity (1). In tumor samples, PD-1 and PD-L1 mainly expressed in T cells and tumor cells, respectively, and tumors exploit their interaction to escape the immune system by counteracting the stimulatory signals from the interaction between T cell receptor (TCR) and major histocompatibility complex (MHC) and other costimulatory signals (2–4).

PD-1/PD-L1 has been translated to the clinical practice, and ICI treatment targeting PD-1/PD-L1 proved to offer significant clinical benefits in many cancers, with an ORR from 20% to 50% in multiple clinical trials and for various types of cancer (5). However, only a small subset of patients showed long-lasting remission, despite remarkable benefits of ICI therapies. Patients of some cancers were completely refractory to checkpoint blockade, occasionally leading to considerable side effects. To predict treatment benefit, PD-L1 expression was proposed as the first biomarker of anti–PD-1/PD-L1 therapy effectiveness (6), followed by tumor mutational burden (TMB) (7). Later, microsatellite instability (MSI) (8), CD8+ T-cell abundance (9, 10), cytolytic activity (11), and intestinal microbial composition (12) were proposed to prioritize patients with potentially more treatment gains.

On the other hand, irAEs result from excessive immunity against normal organs. Most studies show that the incidence of irAEs caused by anti-PD-1/PD-L1 treatment is about 60% (13, 14). Although nearly all organs can be affected, irAEs mostly involved the gastrointestinal tract, endocrine glands, skin, and liver (15). In some cases, irAE can be lethal. For example, pneumonitis is the most common fatal irAE with a 10% death rate, accounting for 35% of anti-PD-1/PD-L1-related fatalities (16). The mortality of myocarditis, the most lethal irAE, could even reach about 50% (17). Therefore, it is important and urgent to select patients with potentially significant benefit over risk of ICI treatments based on individual molecular data.

Although people have discovered several predictors of irAEs using expression of protein-coding genes (18), studying irAE-related non-coding elements would probably provide a better mechanistic understanding of why PD-1/PD-L1 pathway modulation leads to significant clinical benefit in some patients but temporary, partial, or no clinical benefit in other patients.

Recent studies found that eRNAs (non-coding RNAs) were usually transcribed from active enhancers and eRNA levels represent enhancer activities across tissues (19). Numerous cancer-associated eRNAs have been identified and eRNAs were proposed as potential therapeutic targets (20). Here, we comprehensively investigate the adverse events and the response rates in patients receiving anti-PD-1/PD-L1 therapies across cancer types. By integrating clinical data and molecular data, we identified three eRNAs for predicting irAE and another three eRNAs for ORR. Further exploring enhancer-target interaction identified functional genes that may help explain the overall risk or benefit of anti-PD-1/PD-L1 therapy, including MLXIPL, RAF1, MPL, PAK2, DLG1. In summary, our study reveals potential mechanisms underlying ICI therapy based on enhancer activity.

## Results

### Three eRNAs effectively predict irAE of immunotherapy

To identify factors to predict irAEs, we first examined correlations between 7 045 eRNAs and irAE RORs across 25 cancer types. ENSR00000041252 showed the highest correlation (correlation R=0.68, *P*=1.6e-4; **Figure S1A**), stronger than immune factors, including naive B cells, CD8+ T cells, macrophages M1, and T cell receptor diversity (18).

**Figure 1:**
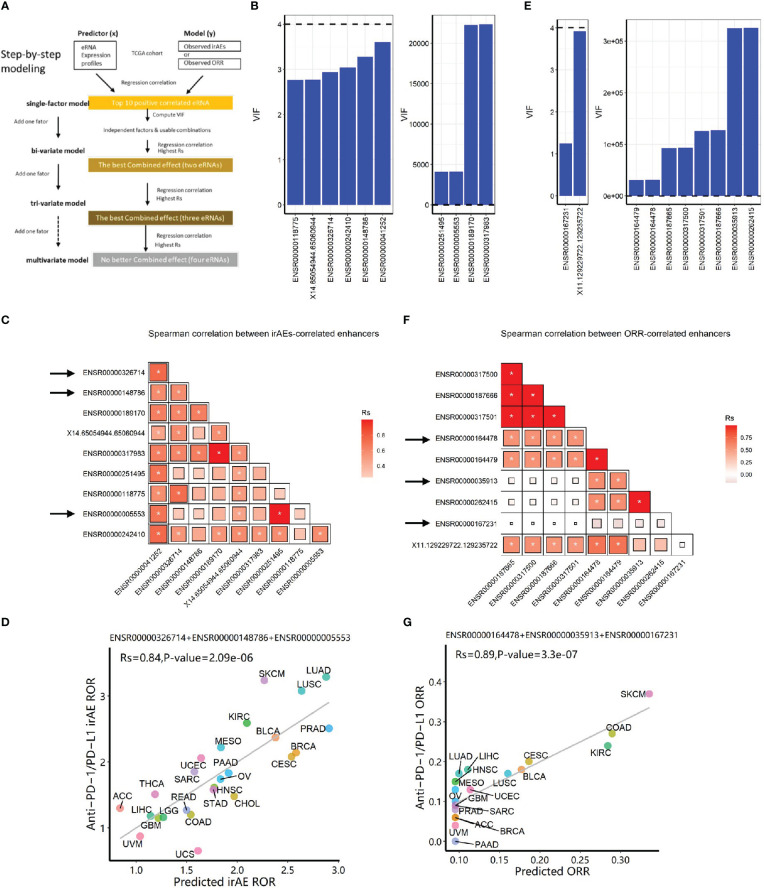
Construction of eRNA-based prediction models for irAE ROR (risk) and ORR (benefit) of immunotherapy. **(A)** The step-by-step workflow of this study. **(B)** Multicollinearity (VIF) analysis for top ten eRNA expression in predicting irAEs. Six eRNAs showed no multicollinearity, while 4 eRNAs showed strong multicollinearity. **(C)** Spearman correlation (Rs) between irAE-correlated eRNAs. The shade of the square indicates the Rs, and the size indicates significance (* indicates statistical significance *P *< 0.05). **(D)** Combined effects of the final trivariate model of predicting irAEs (R=0.84, *P*=2.1e-6). The model is 0.1912*ENSR00000005553+0.4097*ENSR00000326714+0.1953*ENSR00000148786+0.2942. **(E)** Multicollinearity analysis for top ten eRNA expression in predicting ORR. Two eRNAs showed no multicollinearity, while 8 eRNAs showed strong multicollinearity. **(F)** Spearman correlation between ORR-correlated eRNAs. The shade of the square indicates the *Rs*, and the size indicates P-value (* indicates statistical significance *P*< 0.05). **(G)** Combined effects of the final model of predicting ORR (R=0.89, *P*=3.3e-7). The model is 0.0953 + 0.0649*ENSR00000164478+0.0032*ENSR00000035913+0.1687*ENSR00000167231. irAE, immune-related adverse events; ROR, reporting odds ratio; ORR, objective response rates; LUAD, lung adenocarcinoma; SKCM, skin cutaneous melanoma; LUSC, lung squamous cell carcinoma; KIRC, kidney renal clear cell carcinoma; PRAD, prostate adenocarcinoma; BLCA, bladder urothelial carcinoma; MESO, mesothelioma; BRCA, breast invasive carcinoma; CESC, cervical squamous cell carcinoma and endocervical adenocarcinoma; UCEC, uterine corpus endometrial carcinoma; SARC, sarcoma; ESCA, esophageal carcinoma; PAAD, pancreatic adenocarcinoma; OV, ovarian serous cystadenocarcinoma; HNSC, head and neck squamous cell carcinoma; STAD, stomach adenocarcinoma; THCA, thyroid carcinoma; CHOL, cholangiocarcinoma; ACC, adrenocortical carcinoma; READ, rectum adenocarcinoma; COAD, colon adenocarcinoma; LIHC, liver hepatocellular carcinoma; LGG, brain lower-grade glioma; GBM, glioblastoma multiforme; UVM, uveal melanoma; UCS, uterine carcinosarcoma.

Then, we selected the top ten eRNAs with positive correlation and nominal significance (*P*<0.05) (**Table S1**, see **Methods**) to build prediction models, following a step-by-step procedure (**Figure 1A**). Multicollinearity analysis resulted in six roughly independent eRNAs, ENSR00000041252, ENSR00000326714, ENSR00000148786, X14.65054944.65060944, ENSR0000118775, and ENSR00000242410 (**Figure 1B**, **1C**). Next, we obtained 15 significant bivariate regression models using the irAE-correlated enhancers. Correlation between the observed and predicted irAE ROR values showed that two eRNA combinations, ENSR00000148786 + ENSR00000005553 and ENSR00000148786 + ENSR00000251495, achieved the best predictive performance (R=0.79, *P*=3.1e-6; **Figure S1B**). Further increasing model factors resulted in the optimal tri-variate model, ENSR00000326714 + ENSR00000148786 + ENSR00000005553, with the strongest correlation (R=0.84, *P*=2.1e-6; **Figure 1D**). Of note, no improvement was observed after adding the two protein-coding genes (LCP1 and ADPGK) from a model reported previously (18) (**Table S2**). Although showing slightly lower performance than the previous protein-coding gene model (LCP1+ADPGK), our enhancer-based model, explaining 71% (R-squared, R=0.84) of irAE variance, demonstrated that eRNAs alone can effectively predict irAEs.

### Three eRNAs effectively predict immunotherapy benefit

Similarly, we selected the top ten eRNAs with nominal significance (*P*<0.05) of positive correlation with ORRs (**Table S3**, ENSR00000187665 with the highest correlation was shown in **Figure S1C**). After multicollinearity analysis (**Figures 1E, F**), two bivariate models achieved better predictive performance than single-eRNA models (one shown in **Figure S1D**; R=0.82, *P*=2.0e-5). Further adding model factors resulted in four equally-efficient optimal trivariate models (involving five eRNAs, **Table S4**) for ORR prediction were able to effectively predict the efficacy of anti–PD-1/PD-L1 treatments. One example, ENSR00000164478 + ENSR00000035913+ ENSR00000167231, was shown in **Figure 1G** (R=0.89, *P*=3.3e-7).

### eRNA ENSR00000148786 may target TMEM43 to induce irAE during immunotherapy

**Figure 2:**
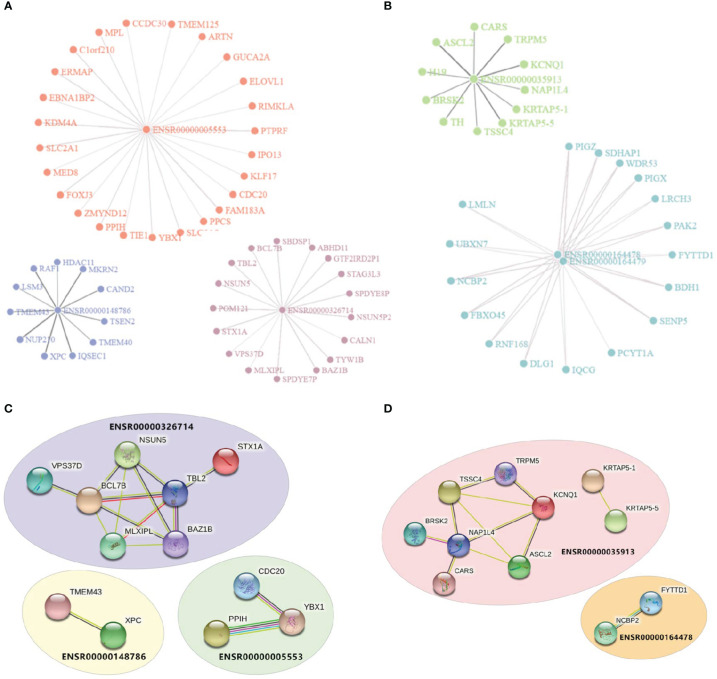
Visualization of enhancer-target interaction network and functional enrichment. **(A)** Target genes of irAE-related enhancers ENSR00000005553, ENSR00000326714, and ENSR00000148786. **(B)** Target genes of ORR-related enhancers ENSR00000164478, ENSR00000164478, and ENSR00000035913. **(C, D)** Protein-Protein Interaction (PPI) networks of target genes of enhancers in the prediction models of irAE **(C)** or ORR **(D)**.

Enhancers were assumed to affect irAEs or ORRs by activating target genes through long-range interactions. We downloaded enhancer-target interaction data (21) and obtained putative targets of our enhancers. Two eRNAs (ENSR00000262415 and ENSRO0000167231) were excluded from downstream analysis due to lack of any annotated target gene. eRNA-target networks showed that these enhancers independently regulated a specific groups of targets (**Figure 2A** for irAE and **Figure 2B** for ORR, note that ENSR00000164478 and ENSR00000164479 located to the same genomic region), indicating that each irAE-related enhancer was involved in different regulatory modules. Similarly, protein-protein interaction (PPI) analysis revealed that an independent network was controlled by each enhancer (**Figures 2C, D**). In these PPI networks, genes located in the center (such as BCL7B, TBL2, and NAP1L4) might be vital regulators of irAEs or ORRs. However, although both BCL7B and TBL2 were closely related to functions of the immune system, no connection between NAP1L4 and immune functions was reported.

**Figure 3:**
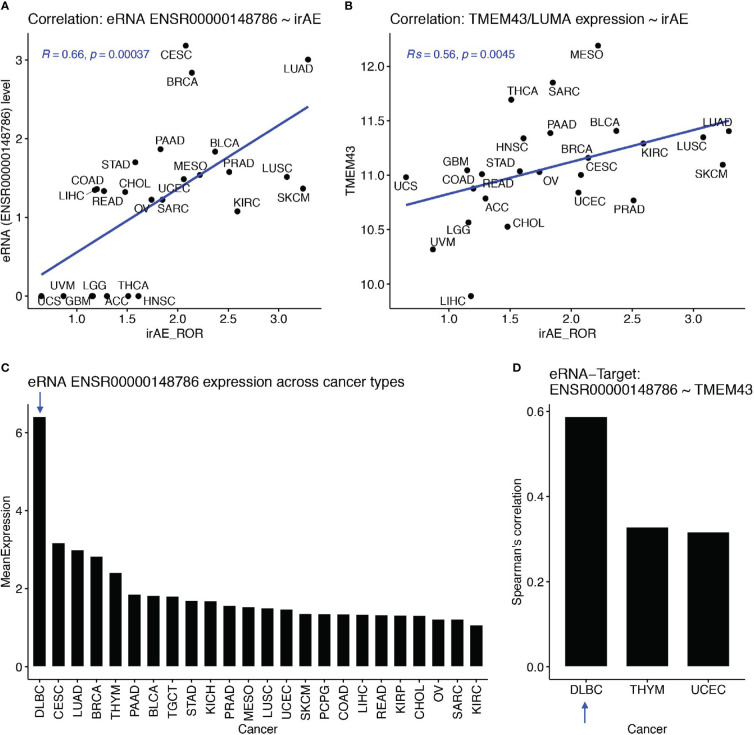
The ENSR00000148786 putative target TMEM43/LUMA potentially induced irAE from B cells. **(A, B)** Significant correlation between irAE and expression levels of eRNA ENSR00000148786 **(A)** or TMEM43/LUMA **(B, C)** Sorted mean expression levels of eRNA ENSR00000148786 across cancer types, with the arrow indicating the highest expression in DLBC (diffuse large B-cell lymphoma). **(D)** Significant eRNA-target correlation between ENSR00000148786 and TMEM43/LUMA was the highest in DLBC among the three significant cancer types.

Then, we examined associations between eRNA targets and irAE or ORR. First, we found that these eRNA-target genes were not among top irAE-related factors reported in the study by Jing et al. (18). We observed the best correlations between SPDYE7P and irAE (R=0.64, *P*=5.1e-4) and between PCYT1A and ORR (R=0.54, P=0.016). After multiple testing correction, only SPDYE7P (speedy/RINGO cell cycle regulator family member E7, pseudogene) and TMEM43 (transmembrane protein 43, also known as LUMA) showed correlation with irAE and with FDR<=0.1. Interestingly, TMEM43/LUMA (chr3:14,124,940-14,143,679), a putative target of irAE-associated ENSR00000148786 (chr3:13,346,900), is known to be able to modulate the innate immune pathways, which can probably induce irAE. Specifically, TMEM43 can form a protein complex with ENDOD1, TMEM33, and TMED1 to promote cGAS-STING signaling (22). It can also activate NF-kB signaling *via* interaction with CARD10 and its associated complex (23). The Spearman correlation between ENSR00000148786 and irAE was 0.66 (P=3.7e-4, **Figure 3A**) and the one between TMEM43/LUMA and irAE was similar (Rs=0.56, P=0.0045, **Figure 3B**).

More importantly, both PD-1 and PD-L1 are critical regulators of B cell functions, which subsequently affect functions of T cells and other immune cells (24, 25). Surprisingly, by using the eRic database (21), we found that ENSR00000148786 expression was the highest in a B-cell malignancy, DLBC (diffuse large B-cell lymphoma) (**Figure 3C**), in which ENSR00000148786 also showed the highest correlation with TMEM43/LUMA (Rs=0.59, FDR= 8.4e-4, **Figure 3D**). It is possible that the much lower levels ENSR00000148786 from tumor types other than DLBC were also originated mainly from tumor-infiltrated B cells in the microenvironment. Although we did not find direct correlations between other eRNA targets and irAE or ORR, those eRNA targets may exert their functions combinatorically.

### Enhancer targets reveal metabolic and inflammatory genes involved in irAEs

**Figure 4:**
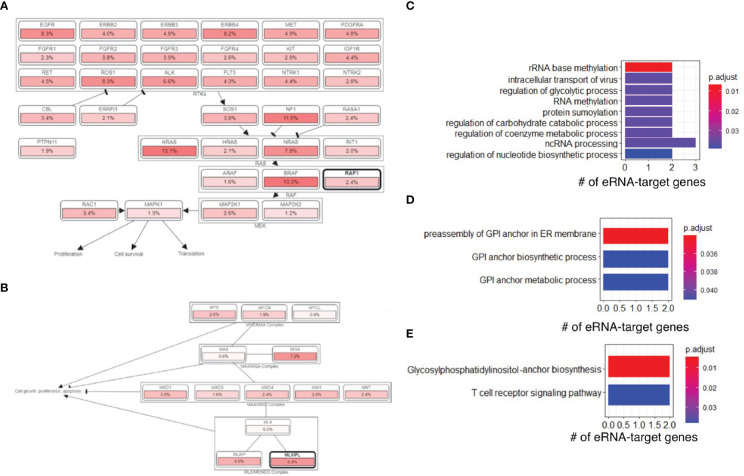
irAE-related genes were involved in oncogenic and other pathways. **(A)** The RAF1 and RTK-RAS signaling pathway. Pathway graphs were generated by using the cBioPortal website. **(B)** The MLXIPL and MYC signaling pathway. **(C)** GO enrichment of genes regulated by irAE-correlated enhancer ENSR00000326714. **(D)** GO enrichment of genes regulated by ORR-correlated enhancer ENSR00000164478. **(E)** KEGG pathway enrichment of genes regulated by ORR-correlated enhancer ENSR00000164478.

Next, we downloaded gene sets from COSMIC (26) and oncoKB (27) and examined our eRNA targets in known oncogenic signaling pathways using cBioPortal (28, 29). We found that some eRNA targets were known cancer genes relevant to tumor immunity, including *MLXIPL, MPL, RAF1*, and *XPC*. *RAF1* was annotated as an oncogene and participated in the RTK-RAS signaling pathway (**Figure 4A**), and MLXIPL was involved in MYC signaling pathway (**Figure 4B**). A previous study (30) showed that RAF1 can activate MAPK1 and NF-κB pathways to regulate genes involved in inflammation. Therefore, RAF1 may enhance immunoreaction and subsequently cause irAEs *via* Natural Killer cell-mediated cytotoxicity, T cell receptor signaling pathway, and B cell receptor signaling pathway, based on functional annotations of RAF1 (**Table S5**).

Interestingly, we found that ENSR00000326714 targets were enriched in a large number of metabolic and biosynthesis processes (**Figure 4C**). This was reminiscent of some types of adverse events, such as diabetes (16), due to metabolic disturbances or metabolic disorders. Specifically, the core network of ENSR00000326714 targets consists of seven metabolic and inflammatory genes, namely, BAZ1B, BCL7B, TBL2, MLXIPL, NSUN, STX1A, and VPS37D. Among them, BAZ1B, BCL7B, TBL2 and MLXIPL are pleiotropic genes for lipids and inflammatory markers in the liver (31). Of note, MLXIPL encodes the carbohydrate-responsive element-binding protein (ChREBP), which mediates glucose homeostasis and liver lipid metabolism. ChREBP was also associated with up-regulation of several cytokines (TNF-α, IL-1β, and IL-6) in patients with type 2 diabetes mellitus, promoting the inflammatory responses and apoptosis of mesangial cells (32). STX1A encodes a member of the syntaxin superfamily, syntaxin 1A. It contributes to neural function in the central nervous system by regulating transmitter release (33). As a kind of target-SNAP receptor (t-SNAREs), it is involved in insulin exocytosis (34). Severely reduced islet syntaxin 1A level was reported to contribute to insulin secretory deficiency (35). Given that diabetes and hepatitis account for ~30% of immune-related adverse events (16), we speculate that ENSR00000326714 probably plays a role in toxic effects in these patients.

### ORR enhancers reveal immune activation genes for immunotherapy benefit

We also analyzed target genes of ORR-predictable eRNAs, which included three types of genes. PAK2, LMLN, DLG1, ASCL2, SENP5, IQCG, and BRSK2 are related to cell cycle, cell division, and differentiation. PIGZ, PIGX, PCYT1A, CARS, and BDH1 are metabolic genes; TRPM5, KCNQ1, and FYTTD1 are responsible for cellular transport and signal transduction. In particular, target genes of ORR-related ENSR00000164478 were enriched in glycosylphosphatidylinositol (GPI)-anchor biosynthesis (FDR=4.73×10^-3^) (**Figure 4D**) and T-cell receptor signaling (FDR=3.78×10^-2^), among other enriched pathways (**Figure 4E**).

Furthermore, PAK2 and DLG1 are involved in the T cell activation pathway (**Table S5**), which may explain their connection with ORR. P21 (RAC1) activated kinase 2 (PAK2) has been reported as a key signaling molecule in the differentiation of T cells. PAK2 is essential in T cell development and differentiation (36), indicating its potential function in T cell-initiated autoimmunity. DLG1 encodes a multi-domain scaffolding protein from the membrane-associated guanylate kinase family, which has been shown to regulate the antigen receptor signaling and cell polarity in lymphocytes, involved in activation and proliferation of T cells (37, 38). Our results provide more support for the T cells as the regulators in immune responses during immune checkpoint blockade therapy. Lastly, PIGZ encodes a protein that is previously identified as an immune-associated prognosis signature (39). However, knowledge of the relationship between PIGZ and the immune system is still poorly established. The association between PIGZ expression and immune benefits during anti-PD1/PDL1 immunotherapy needs further elucidation.

## Discussions

In this work, we presented a preliminary evaluation of the different enhancer-target interactions associated with anti–PD-1/PD-L1 immunotherapy across tumor types, and successfully identified potential enhancer-based biomarkers of risk and beneficial responses. We suggest that, during immunotherapy, enhanced expression of inflammatory factors including TMEM43, BCL7B, TBL2, MLXIPL, STX1A, and RAF1 may lead to a higher risk of irAEs, while immune activation factors including PAK2 and DLG1, in addition to NAP1L4 whose function has not been related to the immune system currently, may improve anti-tumor immunity. Besides, we discovered many other cancer-related, metabolic, signaling or regulatory genes possess predictive potential, which warrants further investigation.

Several limitations remain for future work and our results need to be carefully interpreted. First, the majority of data are collected from previous individual studies (21), introducing inherent limitations of this work. Second, there are inevitable flaws of modeling as well, due to the low expression level of eRNA and small sample size. The overall quality of predictive models of ORR is inferior to those of irAEs, probably due to a smaller sample size as well as larger sparsity of ORR data. Moreover, we only considered eRNAs positively correlated with irAE or ORR. Although further adding eRNAs negatively correlated with irAE or ORR may not further improve model performance and probably cannot compete with mRNA-based models (18), identification of important negatively correlated eRNAs may contribute to the understanding of irAE or ORR mediated by enhancers. Third, another great eRNA and super-enhancer RNA (seRNA) study and its associated database (TCeA), with a much larger number of eRNAs and seRNAs, have been published recently (40), which we hope to integrate into our future work soon. Finally, since results in this project are mainly based on computational predictions and the support of existing literature, our findings need further experimental validation. A larger dataset is required to comprehensively model side effects or immune response as well.

## Methods

### Data collection

To quantify the risk of immune-related adverse events (irAEs), reporting odds ratio (ROR) was calculated as previously described (41). The anti-PD1/PD-L1 irAE ROR and ORR values across different cancer types were collected from previous studies (10, 18). RNA-seq expression data (RSEM normalized counts, log2-transformed) across 25 TCGA cancers were downloaded from the UCSC Xena platform (http://xena.ucsc.edu/). Expression levels of selected genes were extracted for downstream analysis, and the average value was calculated for each TCGA cohort. We downloaded eRNA expression levels and enhancer-target associations for 7 045 enhancer RNAs in ~7,300 samples from the eRic database (21) (https://hanlab.uth.edu/eRic/). Mean eRNA expression (log2-transformed RPM values) were used. Similar to gene expression, we averaged the expression level of each eRNA for each cancer.

### Prediction model construction

FDR control (requiring FDR<0.05) of *P*-values was too strict and resulted in exclusion of all eRNAs from building prediction models. There were mainly two reasons behind this problem. First, most eRNAs had low expression (compared to mRNAs) and many of their estimated expression levels were possibly affected by noise, which severely affected their *P*-values of correlation with irAE (or ORR). Second, the correlation between irAE (or ORR) and eRNAs were based on a small number of summarized data points (only one for each cancer type), further affecting the significance of *P*-values. Therefore, we decided to choose the top ten eRNAs ranked by *P*-values (<0.01) for downstream analysis.

First, the top ten eRNAs were selected based on correlation between eRNA and irAE or ORR. Before constructing bivariate models, the variance inflation factor (42) (VIF) of these ten eRNAs was calculated to evaluate the multicollinearity. Strong multicollinearity indicates redundancy of variables and should be avoided in the prediction models. Generally, we set the threshold of VIF value to 4 (a VIF value greater than 10 will be considered serious multicollinearity). The optimal prediction model was obtained by step-wise addition of model factors (eRNA) and evaluate the correlation between predicted and observed patient risk or benefits.

### Bioinformatics tools

We used the protein-protein interaction (PPI) database STRING (43) (v11, https://string-db.org) to investigate selected eRNA target genes. Basic GO and KEGG term enrichment and visualization were conducted with the R package clusterProfiler (44) (v3.14.3). Extensive functional annotation of eRNA target genes were performed with DAVID (45) (v6.8) (https://david.ncifcrf.gov/). To verify cancer-related function for genes of interest, a credible set of 723 cancer genes was downloaded from the Cancer Gene Census (CGC) project of the COSMIC (26) repository (https://cancer.sanger.ac.uk/cosmic/). Another database oncoKB (27) (https://oncokb.org/), which has a list of 1,064 cancer genes, was added as a supplement to COSMIC CGC genes. Oncogenic signaling pathways were provided by the cBioPortal database (28) (http://www.cbioportal.org/). Statistical analysis and visualization were performed in R (v3.6.3) using packages ggplot2 (v3.3.2), networkD3 (v0.4). For novel candidates, we used three types of biological interpretation (Gene Oncology, Pathways, and Protein-Protein Interaction) to obtain biological knowledge.

### Statistical methods

We employed an approach as described previously (10, 18) to evaluate the correlation between eRNAs and irAE RORs or ORRs. Linear-regression models for predicting irAE ROR or ORR across cancer types, was constructed by the R function lm, and the performance of the prediction was estimated based on Spearman rank correlation, using the R package psych (v2.0.12). To compare the goodness of fit between different models, a log-likelihood ratio test was performed using the R package lmtest (v0.9). We compute variance inflation factor (VIF) to assess multicollinearity using the vif function from the R package car (v3.0) to exclude combinations containing highly correlated factors.

## Data availability statement

The original contributions presented in the study are included in the article/**Supplementary Material**. Further inquiries can be directed to the corresponding author.

## Author contributions

YX and MG conceived and supervised the study. ZL, YX, and MG performed the analysis. MG drafted the manuscript with assistance from ZL. YX reviewed the manuscript. All authors approved the final manuscript. All authors contributed to the article and approved the submitted version.
