# Supplementary material for: Enhancer RNA-based modeling of adverse events and objective responses of cancer immunotherapy reveals associated key enhancers and target genes
Source: Front Oncol. 2023 Jan 19;12:1048127. doi: 10.3389/fonc.2022.1048127 (PMC9893284; doi:10.3389/fonc.2022.1048127)

**Supplementary Information**

# Supplementary Tables

**Table S1. Top ten significant eRNAs associated with irAE.**

| **eRNA** | **R** | ***P*-value** |
| --- | --- | --- |
| ENSR00000041252 | 0.68 | 1.64E-04 |
| ENSR00000326714 | 0.66 | 3.17E-04 |
| ENSR00000148786 | 0.66 | 3.75E-04 |
| ENSR00000189170 | 0.62 | 8.95E-04 |
| X14.65054944.65060944 | 0.62 | 1.01E-03 |
| ENSR00000317983 | 0.62 | 1.06E-03 |
| ENSR00000251495 | 0.61 | 1.31E-03 |
| ENSR00000118775 | 0.60 | 1.46E-03 |
| ENSR00000005553 | 0.60 | 1.59E-03 |
| ENSR00000242410 | 0.60 | 1.62E-03 |

**Table S2.** Performance comparisons of irAE models combining known coding genes and eRNAs. (x2: ENSR00000326714, x3: ENSR00000148786, x9: ENSR00000005553)

| Enhancer  model | Coding gene  model | Rs | P-value | Log likelihood ratio test vs enhancer | Log likelihood ratio test vs coding |
| --- | --- | --- | --- | --- | --- |
| x2+x3+x9 | ADPGK | 0.87 | 2.23E-06 | 0.15 | 0.00 |
| x2+x3+x9 | LCP1 | 0.86 | 2.22E-06 | 0.19 | 0.02 |
| x2+x3+x9 | LCP1+ADPGK | 0.88 | 2.22E-06 | 0.21 | 0.06 |

**Table S3. Top ten significant eRNAs associated with ORR.**

| **Factor name** | **eRNA** | **R** | ***P*-value** |
| --- | --- | --- | --- |
| *x1* | ENSR00000187665 | 0.59 | 7.46E-03 |
| *x2* | ENSR00000317500 | 0.59 | 7.46E-03 |
| *x3* | ENSR00000187666 | 0.55 | 1.37E-02 |
| *x4* | ENSR00000317501 | 0.55 | 1.37E-02 |
| *x5* | ENSR00000164478 | 0.51 | 2.44E-02 |
| *x6* | ENSR00000164479 | 0.51 | 2.44E-02 |
| *x7* | ENSR00000035913 | 0.51 | 2.45E-02 |
| *x8* | ENSR00000262415 | 0.51 | 2.45E-02 |
| *x9* | ENSR00000167231 | 0.50 | 2.76E-02 |
| *x10* | X11.129229722.129235722 | 0.50 | 2.78E-02 |

**Table S4. Combined effects of the best trivariate ORR models.** *x5* ENSR00000164478, *x6* ENSR00000164479, *x7* ENSR00000035913, *x8* ENSR00000262415, *x9* ENSR00000167231.

| **Trivariate model** | **Rs** | **P-value** |
| --- | --- | --- |
| *x5+x7+x9* | 0.89 | 3.31E-07 |
| *x6+x7+x9* | 0.89 | 3.31E-07 |
| *x5+x8+x9* | 0.89 | 3.00E-07 |
| *x6+x8+x9* | 0.89 | 3.00E-07 |

**Table S5.** DLG1, PAK2, and RAF1 are involved in immune-related functions. Functional annotations are from the DAVID database.

| **Symbol** | **Description** | **GOTERM_BP_DIRECT** | **KEGG_PATHWAY** |
| --- | --- | --- | --- |
| DLG1 | discs large MAGUK scaffold protein 1 | T cell activation (GO:0042110), negative regulation of T cell proliferation (GO:0042130) | T cell receptor signaling pathway (hsa04660) |
| PAK2 | p21 (RAC1) activated kinase 2 | T cell receptor signaling pathway (GO:0050852), T cell co-stimulation (GO:0031295) | \ |
| RAF1 | Raf-1 proto-oncogene, serine/threonine kinase | \ | Natural killer cell mediated cytotoxicity(hsa04650), T cell receptor signaling pathway(hsa04660), and B cell receptor signaling pathway(hsa04662) |

# Supplementary Figures

**Fig. S1. Correlation between eRNAs and irAE or ORR.**

(**A**) Spearman correlation between ENSR00000041252 expression and irAE ROR (R=0.68, *P*=1.6e-4). (**B**) Combined effect of ENSR00000148786 and ENSR00000251495 bivariate model of predicting irAEs. (R=0.79, P=3.08×10^−6^). The equation of the best bivariate model is 0.3732* ENSR00000148786+0.2181*ENSR-00000251495+1.2144. (**C**) Spearman correlation between ENSR00000187665 expression and ORR (R=0.59, *P*=7.5e-3). (**D**) Combined effect of ENSR00000035913 and ENSR00000167231 bivariate model of predicting ORR (R=0.82, *P*=2.0e-5). The equation of the best bivariate model is 0.0063 * ENSR00000035913+0.1596 * ENSR00-000167231+0.1070.


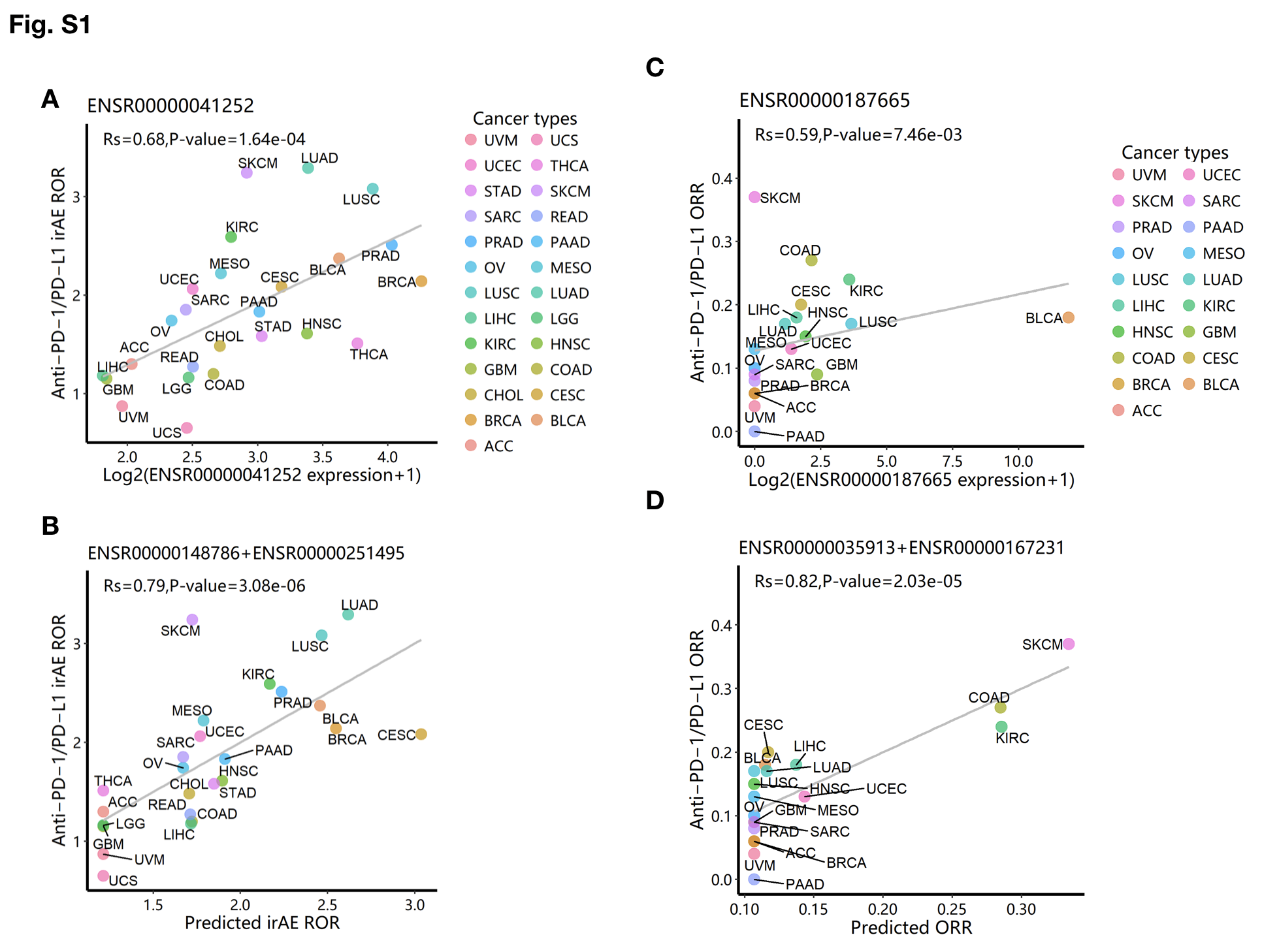

Supplement: Supplementary file 1 [file DataSheet_1.docx]
